# Multiplanar AS-OCT Detection of Clinically Occult Posterior Gas Bubble Dislocation After DSAEK

**DOI:** 10.3390/diagnostics16091267

**Published:** 2026-04-23

**Authors:** Wojciech Luboń, Małgorzata Luboń, Mariola Dorecka

**Affiliations:** 1 Department of Ophthalmology, Faculty of Medical Sciences, Medical University of Silesia, 40-514 Katowice, Poland; mdorecka@sum.edu.pl; 2 Department of Ophthalmology, Professor K. Gibiński University Clinical Center, Medical University of Silesia, 40-514 Katowice, Poland; kozikowskamalg@gmail.com

**Keywords:** DSAEK, anterior segment OCT, endothelial keratoplasty, bullous keratopathy, gas tamponade, SF6, malignant glaucoma, postoperative complications, slit-lamp examination

## Abstract

Descemet stripping automated endothelial keratoplasty (DSAEK) is a well-established surgical technique for the treatment of endothelial dysfunction, in which intracameral gas tamponade plays a critical role in graft adherence. We report the case of a 67-year-old pseudophakic woman with advanced Fuchs endothelial corneal dystrophy and symptomatic pseudophakic bullous keratopathy in the right eye, who presented with progressive visual deterioration and underwent DSAEK using an 8.25 mm donor graft inserted with a Busin glide and tamponaded with a 25% sulfur hexafluoride (SF6) gas–air mixture. On the first postoperative day, slit-lamp examination suggested an appropriate anterior chamber configuration and satisfactory graft attachment. However, detailed multiplanar anterior segment optical coherence tomography (AS-OCT), defined here as assessment using vertical, horizontal, and rotational scan orientations, revealed subtle posterior migration of the gas bubble beneath the iris plane. This clinically occult finding indicated altered anterior segment anatomy associated with a risk of secondary angle-closure mechanisms and raised concern for malignant glaucoma. Prompt surgical re-intervention was undertaken on postoperative day one, involving decompression of the misdirected gas bubble and reinjection of a centrally positioned tamponade. This resulted in restoration of normal anterior chamber configuration and stable graft adherence. Best-corrected visual acuity (BCVA) improved from 0.1 Snellen (1.0 logMAR) preoperatively to 0.7 Snellen (0.15 logMAR) at 2 weeks following surgery. This case highlights the added value of multiplanar AS-OCT in detecting clinically occult posterior gas migration after DSAEK, particularly when the abnormality is scan-orientation-dependent and not apparent on slit-lamp examination, thereby enabling timely intervention in the presence of a potentially sight-threatening postoperative configuration.

**Figure 1:**
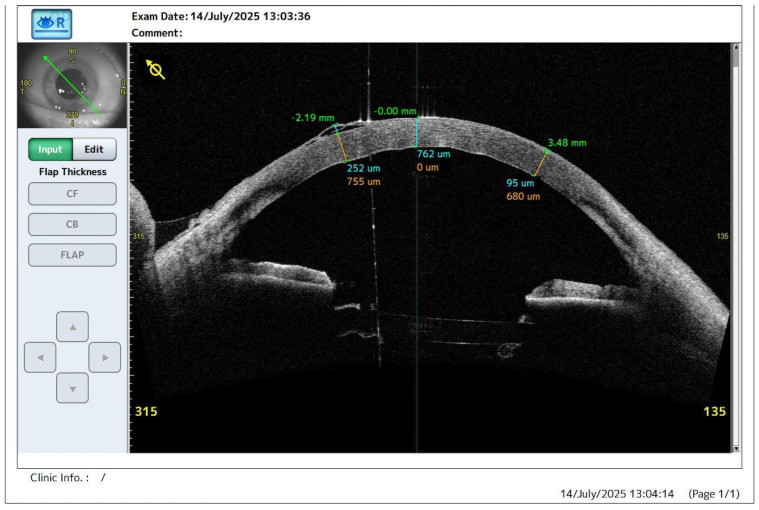
Preoperative anterior segment optical coherence tomography (AS-OCT; CASIA2, Tomey Corporation, Nagoya, Japan) demonstrating advanced corneal edema consistent with symptomatic pseudophakic bullous keratopathy secondary to Fuchs endothelial corneal dystrophy in the right eye of a 67-year-old woman. The patient had undergone uncomplicated cataract surgery in the affected eye 3 years earlier and presented with progressive visual deterioration over approximately 6 months, accompanied by ocular pain, foreign-body sensation, photophobia, stinging, and discomfort related to recurrent epithelial bullae. Specular microscopy showed reduced endothelial cell density of 690 cells/mm^2^ with marked polymegathism and pleomorphism, consistent with advanced endothelial dysfunction. Systemic comorbidities included arterial hypertension, anemia, gout, and generalized atherosclerosis. Before surgery, she had been treated with 5% sodium chloride hypertonic eye drops five times daily, 5% sodium chloride ophthalmic ointment at night, and dexamethasone eye drops twice daily for 1 month; however, the therapeutic effect gradually diminished and symptoms progressed. AS-OCT shows marked stromal edema with central corneal thickness of 762 µm and epithelial detachment with multiple subepithelial bullae. Preoperative best-corrected visual acuity (BCVA) was 0.1 Snellen (1.0 logMAR), and intraocular pressure (IOP) measured 18 mmHg. Due to persistent symptoms, failure of conservative treatment, and declining visual function, the patient was qualified for Descemet stripping automated endothelial keratoplasty (DSAEK). Endothelial keratoplasty is an established treatment option for corneal endothelial decompensation, including cases associated with pseudophakic bullous keratopathy [1].

**Figure 2:**
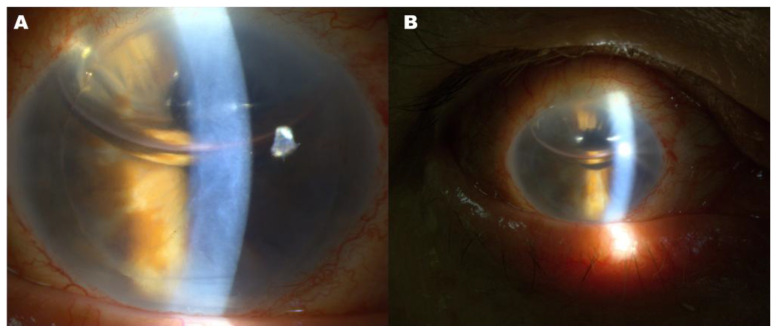
Early postoperative slit-lamp imaging with clinically unapparent gas misdirection. (**A**,**B**) Slit-lamp photographs obtained 6 h after DSAEK show a large intracameral gas bubble with typical superior positioning. Surgery was performed using an 8.25 mm donor graft inserted with a Busin glide. Continuous irrigation of the anterior chamber was maintained during graft insertion, and corneal tapping was used to facilitate appropriate graft positioning and adherence. At the end of the procedure, the anterior chamber was completely filled with a 25% sulfur hexafluoride (SF6) gas–air mixture for 15 min, after which the bubble was reduced to approximately 50% of the anterior chamber volume. The graft appeared well apposed, and the anterior chamber configuration seemed clinically appropriate. However, posterior gas migration behind the iris was not detectable on slit-lamp examination, highlighting the limitation of clinical assessment alone in identifying subtle postoperative gas misdirection [2,3].

**Figure 3:**
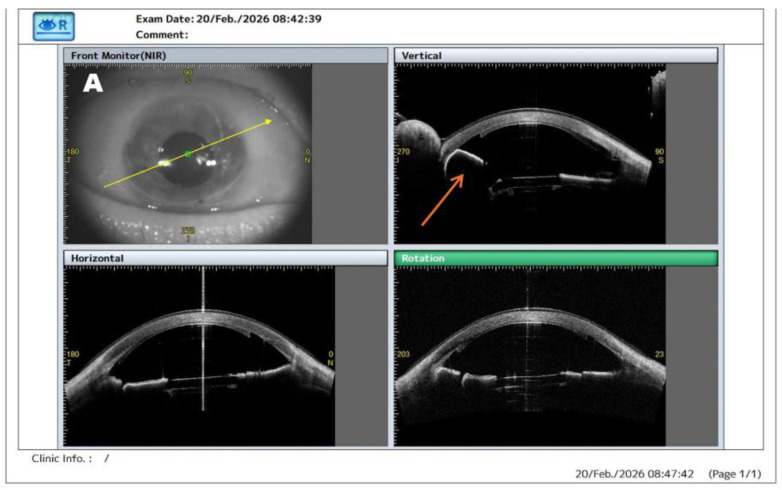

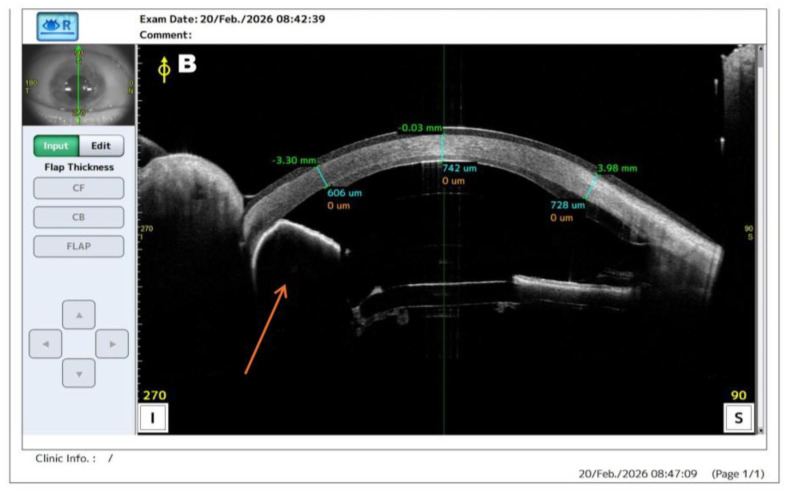
AS-OCT detection of clinically occult posterior gas migration after DSAEK. (**A**) AS-OCT assessment on postoperative day one was performed using vertical, horizontal, and rotational scan orientations centered on the pupil and anterior chamber. Posterior displacement of the gas bubble beneath the iris is visible only in the vertical scan (arrow), whereas no abnormality is identified in the horizontal and rotational sections, indicating its localized and orientation-dependent nature. Notably, this finding was not apparent on slit-lamp examination (Figure 2), and IOP at that time was 20 mmHg, indicating that the abnormality was detected before overt clinical decompensation. (**B**) Magnified vertical AS-OCT image highlighting posterior migration of the gas bubble beneath the iris plane (arrow), associated with anterior displacement of the iris–lens diaphragm and early shallowing of the anterior chamber. The arrow indicates the site of gas misdirection. Based on the AS-OCT findings, prompt surgical re-intervention was performed with decompression of the misdirected gas bubble and reinjection of a centrally positioned tamponade. Although posterior gas migration after endothelial keratoplasty has been previously described [4], this case highlights the additional value of multiplanar AS-OCT in detecting a clinically occult and scan-dependent postoperative abnormality that may otherwise be overlooked, consistent with the broader role of AS-OCT in the postoperative assessment and management of endothelial keratoplasty [5,6]. Such early anatomical recognition is clinically relevant because these changes may precede IOP elevation, secondary angle closure, and graft compromise [7,8].

**Figure 4:**
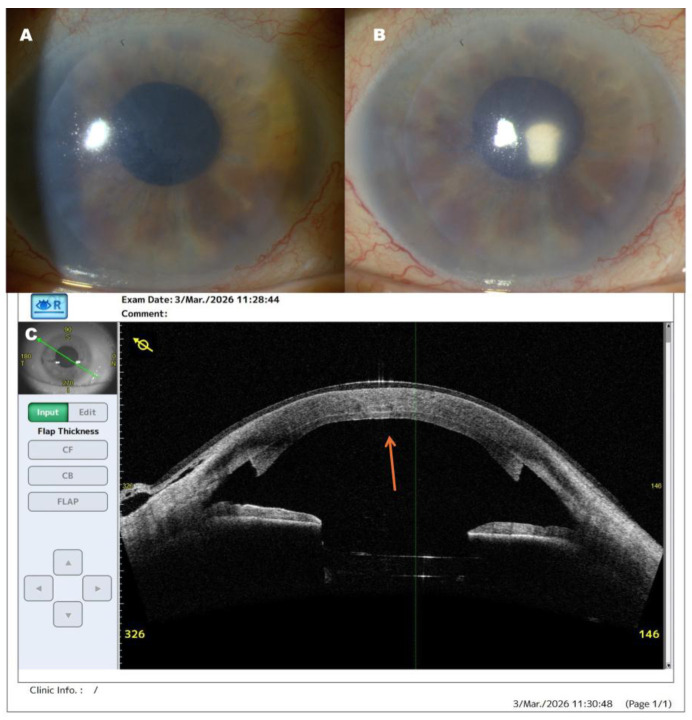
Postoperative outcome at 2 weeks after DSAEK. (**A**,**B**) Slit-lamp photographs obtained 14 days after surgery demonstrate a well-centered and fully attached endothelial graft with marked improvement in corneal clarity. The optical zone shows progressive transparency with regression of stromal edema. No signs of graft detachment, interface opacities, or immunologic rejection are observed. The anterior chamber remains well formed, without evidence of shallowing or abnormal iris configuration. BCVA at this stage was 0.7 Snellen (0.15 logMAR). (**C**) AS-OCT confirms complete graft adherence with a regular interface and absence of interface fluid; the arrow indicates the fully attached and well-centered endothelial graft, consistent with the established utility of AS-OCT in evaluating postoperative graft morphology after endothelial keratoplasty [6]. The postoperative image demonstrates restoration of a regular anterior segment configuration after re-intervention, without imaging features suggestive of persistent postoperative abnormality. Timely recognition and appropriate management of early postoperative anterior segment abnormalities are essential for maintaining graft attachment, preserving visual outcomes, and reducing the risk of secondary complications after endothelial keratoplasty [1,5]. For clinicians, this case emphasizes the importance of careful postoperative follow-up and a low threshold for additional anterior segment imaging when the clinical picture and anatomical configuration are not fully concordant, as prompt correction of subtle postoperative abnormalities may help preserve graft integrity and prevent further adverse sequelae [3,8].

## Data Availability

The original contributions presented in this study are included in the article. Further inquiries can be directed to the corresponding author.

## Abbreviations

The following abbreviations are used in this manuscript:

AS-OCT	Anterior Segment Optical Coherence Tomography
BCVA	Best-Corrected Visual Acuity
DSAEK	Descemet Stripping Automated Endothelial Keratoplasty
IOP	Intraocular Pressure
